# The human microbiome, asthma, and allergy

**DOI:** 10.1186/s13223-015-0102-0

**Published:** 2015-12-10

**Authors:** Amund Riiser

**Affiliations:** Faculty of Teacher Education and Sports, Sogn og Fjordane University College, Sogndal, Norway

**Keywords:** Asthma, Allergy, Microbiome

## Abstract

The human microbiome can be defined as the microorganisms that reside within and on our bodies and how they interact with the environment. Recent research suggests that numerous mutually beneficial interactions occur between a human and their microbiome, including those that are essential for good health. Modern microbiological detection techniques have contributed to new knowledge about microorganisms in their human environment. These findings reveal that the microbiomes of the lung and gut contribute to the pathogenesis of asthma and allergy. For example, evidence indicates that the microbiome of the gut regulates the activities of helper T cell subsets (Th1 and Th2) that affect the development of immune tolerance. Moreover, recent studies demonstrate differences between the lung microbiomes of healthy and asthmatic subjects. The hygiene and biodiversity hypotheses explain how exposure to microorganisms is associated with asthma and allergy. Although those living in developed countries are exposed to fewer and less diverse microorganisms compared with the inhabitants of developing countries, they are experiencing an increase in the incidence of asthma and allergies. Detailed analyses of the human microbiome, as are being conducted under the auspices of the Human Microbiome Project initiated in 2007, promise to contribute insights into the mechanisms and factors that cause asthma and allergy that may lead to the development of strategies to prevent and treat these diseases.

## Background

Microbiomes exist in numerous diverse environments such as soil, freshwater, and seawater. The human microbiome was studied for the first time in the 1600s when Antonie van Leeuwenhoek scraped the coating of his teeth and studied the contents using his self-fabricated microscope [1]. Just over 300 years later, the Nobel Laureate Joshua Lederberg coined the term “the human microbiome” to describe the ecological community of symbiotic and pathogenic microorganisms that inhabit the body [2]. Lederberg believed that microorganisms in the human body are significant for health and disease [2]. Scholars distinguish between the concepts of microbiome [3, 4] and microbiota [5] to separate the collective genomes of microorganisms from the microorganisms themselves, although the original definitions do not distinguish between them [3–5].

An adult human harbors approximately 100 billion bacteria in the intestine alone, and the microbiome accounts for 90 % of the cells in the human body [3]. The human genome comprises about 21,000 genes that encode proteins [6]. In contrast, the microbiome may comprise approximately three million genes [4]. The microbiome may be considered a “new organ system,” because its existence and contributions to human health and disease was uncovered by researchers between 15 and 20 years ago [7]. The composition and function of the microbiome of the human gut evolves during the first years of life and stabilize within the first 3 years of life [8–10]. The development of the gut microbiome is influenced by interactions between diet, environment, and host- and microbe-associated factors [10–18]. The gut microbiome plays a fundamental role in shaping host immunity by balancing the activities of Th-1 cells and Th-2 cells [19–23]. In contrast to the extensive knowledge of the gut microbiome [11, 24], information on the lung microbiome is limited [25, 26]. However evidence suggests a distinct microbiome of the lungs of healthy subjects [27] and a difference between the microbiomes of healthy people and those with obstructive lung diseases such as asthma [28].

The aim of the present review was to provide a general account of current research on the gut and lung microbiomes of humans and their association with the pathogenesis of allergy and asthma.

## Review

### The human microbiome

Over the last 30 years, the development of techniques to sequence microbial ribosomal RNA genes has led to the reconstruction of the evolutionary history of microorganisms [29, 30] and the illumination of their ecology. The United States National Institutes of Health initiated the Human Microbiome Project (HMP), which was allocated a budget of 150 million USD to investigate the microbiota of the human nose, mouth, gut, skin, and genitourinary tract with the aim of documenting the changes in the microbiome associated with human health [4]. Contributions of the HMP include the discovery that the microbiomes of the anatomic sites described above are similar among individuals but vary between the hair, nose, mouth, gut, skin, and genitourinary tract [24]. However, there may be large individual variations in microbiomes at the same sites on the body among individual [31].

### The gut microbiome

The most thoroughly studied microbiome is that of the human gut [26]. Swift adaptation to modern lifestyles and environmental transitions likely changed the human gut microbiome with clear effects on immunological and physiological processes that affect human health [11]. The stability and diversity of the gut microbiome increase over the first 3 years of life [8–10]. Perinatal exposure [14], gestational age [16], mode of delivery [12], host genetics [15], and breastfeeding [10, 13] influence the development of the microbiomes of infants while antibiotics [18] and diet may also influence the microbiomes of older people [17]. *Firmicutes* and *Bacteroidetes* dominate the gut microbiome of adults; however, the representation and diversity of different classes of bacteria vary enormously [4].

A recent study of fecal samples of 124 European subjects identified between 1000 and 1150 bacterial species. Each individual in the cohort harbored at least 160 bacterial species, many of which were shared [32]. Knowledge gained from research on the gut microbiome provides insight into the development of allergic diseases and why the far less studied lung microbiome may contribute to the development of asthma. Factors such as birth by cesarean section [12, 33], breastfeeding [33], introduction of solid foods [10], and use of antibiotics by the mother or infant [34] affect the gut microbiome and are associated with increased incidences of asthma and allergies [35]. For example in a study including 2733 1-month-old infants, colonization of with *Clostridium difficile* is associated with birth by cesarean section and an increase in the risk of asthma (aOR = 2.06) at 6 years of age [36]. However, there are great inconsistencies in the findings of studies on the association between delivery by caesarian section and proven food allergy [37].

However, there is persuasive evidence indicating that children born by cesarean section have twice the risk of being sensitized to egg and milk allergens [38]. A recent study [40] of the association between gut microbiota and food sensitization in the first year of life among 166 infants found that 7.2 % were sensitized to one or more food allergens at 1 year of age and had lower gut microbiota richness and an elevated *Enterobacteriaceae*:*Bacterioidaceae* ratio. These findings suggest that early gut colonization may contribute to the development of atopic diseases such as food allergy [39]. Interaction between the host and the gut microbiome may explain the relationship between the microbiome and asthma and allergies.

Studies conducted using mice show that the gut microbiome is important in shaping the host’s immune system [23]. Mice with different genetic predispositions to polarize responses to different antigens in opposite directions, toward either Th1 cells (possibly suppressed in asthma and allergy [40]) or Th2 cells (enhanced in asthma and allergy [40]) depending on the stimulus, both have airway response when the microbiome is disrupted [41]. By controlling the activation of antigen-presenting dendritic cells in the gut, single bacterial species may regulate the differentiation of naïve T cells into regulatory T cells [20]. The microbiome may affect the balance between Th1 cells and Th2 cells and thus the outcomes of subsequent infections with pathogens [20]. This is just one of many examples of immune regulation in symbiosis with bacteria [17, 19]. Further, specific bacterial species directly influence the development of regulatory T cells in mice [21] and humans [22]. Thus, symbiotic bacteria may regulate the immune system.

### The lung microbiome

Researchers believed mistakenly for many years that the lungs are sterile [42]. For example, in a study of 28 healthy adults, the most common phyla detected in the nose and oropharynx are *Actinobacteria*, *Bacteroidetes*, *Firmicutes*, and *Proteobacteria* and that 2000 bacterial genomes per cm^2^ are present in the upper left lobe of the lung [26]. *Proteobacteria*, particularly *Haemophilus* spp., are more common in the lungs of asthmatic adults than in those of healthy controls who harbor a higher proportion of *Bacteroidetes*. Further, the abundance of *Proteobacteria* in asthmatic children is higher compared with healthy controls [28].

In children with asthma, the bacterial load is significantly higher in the lung compared with healthy controls [44]. Further, Airway microbiological diversity in the lungs was significantly greater in asthmatic children who demonstrated significant reduction in bronchial responsiveness after antibiotic treatment [43]. Six weeks of treatment of patients with asthma with azithromycin reduces the relative abundance of *Prevotella* from 4.54 to 3.43 %, *Staphylococcus* from 10.49 to 4.59 %, and *Haemophilus* from 10.74 to 3.28 %. In some patients treatment with azithromycin reduces the bacterial richness in the airways and *Anaerococcus becomes* the most abundant bacteria. [44]. Further research is required to understand the role of the lung microbiome in the pathogenesis of asthma.

### The microbiome in allergy and asthma

Increasing evidence suggests that the compositions of the lung and gut microbiomes determine the risk of asthma and allergies. It is interesting to consider how these findings support or contradict the “hygiene” and “biodiversity” hypotheses (see below) that were proposed to account for the influence of the human microbiota on allergies and immune tolerance. The hygiene hypothesis argues that the lack of early exposure to infectious agents, symbiotic microorganisms (such as intestinal flora and probiotics), and parasites increases susceptibility to allergic diseases through insufficient stimulation of Th1 cells [45], which cannot counterbalance the effects of Th2 cells, leading to predisposition to allergic diseases [20]. The biodiversity hypothesis suggests that disturbances in the composition of the gut microbiome of citizens of western countries induced by antibiotics, diet, and lifestyle disrupt the mechanisms of mucosal immunological tolerance [46]. The biodiversity hypothesis extends the hygiene hypothesis by stating that the gut microbiome interacts with the immune system to maintain the efficiency of the immune system [47].

### The hygiene hypothesis

The hygiene hypothesis was proposed to explain the invers association between the risk of hay fever and the number of older siblings [46]. Thus, infections in early childhood transmitted through unhygienic contact with older siblings, or through the mother who is infected by her older children, prevent the development of allergic disease [45]. This hypothesis is supported by the findings of a study on school children in areas of Germany that were formerly East and West Germany that showed a decreased risk of allergic sensitization with an increasing number of older siblings, which was indicated by a rate of allergic sensitization this was three times higher in the former West compared with East Germany [48]. Numerous subsequent cross-sectional studies support the hygiene hypothesis, because they show that increased exposure to bacteria, fungi, airway infections, dogs, cats, and other animals in childhood is associated with decreased risk of asthma and allergies [49, 50]. Other studies show a lower incidence of allergic diseases in children raised on farms [51].

The hygiene hypothesis has been “updated” based on cross-sectional data together with experimental data and can be reconciled by a “new hygienic hypothesis” or a “Western lifestyle hypothesis,” [52] which proposes that Th1 cells are not stimulated to balance the Th2 cells. Imbalance in the Th2 cell population leads to the production of interleukins (IL-4 and IL-5), which then induce the production IgE and eosinophils that cause atopic disease [53]. The relationship between exposure to different types of bacteria or fungi and asthma in children was assessed in a review of the characteristics of more than 15,000 children included in two different studies [50]. This analysis found that children who lived on a farm are exposed to diverse microflora compared to children not living on farms. The two studies analyzed show a reduced prevalence of asthma in children who live on a farm compared children not living on farms and an inverse association between exposure to microbes and the probability of asthma. However, a correlation between exposure to the microbiota and antigen-specific IgE was not detected [49].

Because cross-sectional studies simultaneously measure exposure and effect, they cannot establish a causal relationship. For example, the findings of cross-sectional studies may be explained if the parents of children with atopic disease have fewer children compared with other parents. Thus, the lower incidence of asthma and allergies in people who live on farms may be explained by families with asthma who discontinue farming and move; in contrast, healthy families continue to farm. Such an effect is called the healthy worker effect [54]. Occupational epidemiology studies suggest that the healthy worker effect reduces the association between exposure and outcome by 20–30 % [55] and that the effect of “cat avoidance” could correspond to a protective effect with an odds ratio of 0.83 [56]. Several longitudinal studies did not detect similar effects that support the hygiene hypothesis [57, 58], and several studies of birth cohorts show an positive association between exposure to cats and cat allergen-specific IgE [59, 60]. Other observations such as increased atopy in poor and densely populated urban areas and among children in kindergarten argue against the hygiene hypothesis [61].

### The biodiversity and the microflora hypotheses

Biodiversity is important for human livelihood and development and plays a paramount role in the quality of life of populations worldwide [62]. Moreover, the loss of biodiversity may have serious implications for human health [63]. Evidence indicates that the diversity of the body’s microbiota is negatively associated with the risk of developing asthma and allergy [46, 64]. The biodiversity and disappearing microbiota hypotheses argue that changes in how people interact with the environment reduces exposure to microorganisms and that this may affect the mechanisms of development of immunologic tolerance [46, 65]. For example, consumption by mothers and infants of untreated cow’s milk is negatively associated with asthma and allergies in children regardless of whether they live on a farm [66]. Further, a diverse intestinal flora in early life is associated with reduced risk of allergy at 5 years of age [67], and the diversity of gram-negative *Gammaproteobacteria*, common in soil but particularly dominant in aboveground vegetation, is reduced on the skin the atopic adolescents [68]. The association of gram-negative *Gammaproteobacteria* such as *Acinetobacter* with atopic disease is supported by the positive correlation of the levels of a regulator of immune tolerance, IL-10, with the abundance of *Acinetobacter* on the skin of healthy but not atopic adolescents [68].

The microflora hypothesis extends the hygiene and biodiversity hypotheses by proposing that the gut microbiome interacts with the immune system to maintain immune function. An imbalance in the gut microbiome caused by changes in the use of antibiotics and in the diet over the last three decades may cause dysfunction of the immune system [47]. This may explain why diseases such as asthma and allergy develop at any age. The evidence supporting the microflora hypothesis includes the increased incidences of asthma and allergies in industrialized countries during the last 50 years [69, 70] and includes the major relationships as follows: (1) correlation between allergic diseases and antibiotic use [71, 72], (2) correlation between allergic diseases and altered fecal microbiota [73, 74], and (3) correlation between allergic diseases and dietary changes [75]. Studies of mice demonstrate that antibiotic treatment that disrupts the microbiome may inhibit airway tolerance to airborne allergens such as fungal spores (e.g. *Aspergillus fumigatus*) [76] or aerosolized ovalbumin [41].

### Probiotics

Probiotics are defined as viable microorganisms that enhance the host’s health [77]. There is great uncertainty about the efficacy of probiotics for preventing and treating asthma and allergies. For example, when *Lactobacillus* was administered prenatally to mothers with at least one relative or partner with allergic rhinitis, atopic eczema, or asthma, and postnatally for 6 months to their infants, the outcome indicated a promising effect on the prevention of atopic disease [78]; however, the results of more recent studies (mainly of *Lactobacillus* and *Bifidobacterium*) are inconsistent [79]. A meta-analysis of 25 studies that assessed the effects of probiotic administration in children on atopy and asthma found that administration of probiotics reduces IgE levels and the risk of atopic sensitization but not the risk of asthma or wheezing [80]. The timing of ingestion, prenatally to the mother and postnatally to the infant versus postnatally to only the infant, did not influence IgE levels; however, the risk of allergic sensitization was significantly reduced only when the administration of probiotics commenced prenatally and continued after birth. The decrease in total IgE was more pronounced when probiotics were administered for longer, and the reduced risk of allergic sensitization may therefore depend on the specific bacterial strains. These findings indicate that administration of probiotics during pregnancy and to infants may contribute to the prevention of atopic diseases in high-risk infants [80].

Bacterial products may have great potential for preventing and treating allergies, although the bacterial species, their numbers, and the duration and timing of treatment that are safe and effective are unknown [46]. The results of a controlled clinical trial of newborns indicate that 6 months of treatment with oral probiotics protects against IgE-associated dermatitis at 2 years, but only for those at high risk of eczema. The effect disappears after 5 years of age [81]. Future probiotic supplements are likely to contain a wide range of microbes that can have long-term beneficial effects on the immune system [82]. Therefore, immunological, epidemiological, microbiological, and clinical studies are required to establish whether supplementation with bacterial products contribute to effective treatment and prevention of asthma and allergy. Future trials investigating the effects of probiotics should include analyses of specific strains of probiotic bacteria and longer follow-up.

### Areas for future exploration

Allergy and asthma are heterogeneous diseases but are categorized according to an increased tendency of inflammation driven by changes in the environment, nutrition, the gut microbiome, and possibly the lung microbiome. The outcome of these changes may depend on genotype [83, 84], overall biodiversity [46], and other risk factors such as physical activity [85] and air pollution [86]. The findings may contribute a common explanation of why the incidence of numerous inflammatory conditions such as asthma and allergy has increased in parallel with changes in the environment [11]. A broad interdisciplinary approach will be required to understand the complex pathogenesis of asthma and allergy and to understand the roles of the lung and gut microbiomes. The use of standardized outcomes and methodology may help address the gaps in our knowledge [11, 87]. For example, researchers should continue to focus on the functional and ecologic characteristics of the gut and lung microbiomes of healthy people as well as the features of the lung and gut microbiomes of patients with specific diseases. The development of strategies to improve microbiome colonization patterns will likely enhance health throughout an individual’s life. It will be necessary to determine if variations in the microbiome are the cause or effect of allergies and asthma, and longitudinal studies are essential to control for different confounding factors. Further research on the microbiome should include organisms other than bacteria as some viruses [88] and fungi [89] interact with their hosts cells similar to what’s observed with bacteria and their host. The results of such research can be ultimately translated to the clinic to improve diagnostics as well as treatment and prevention strategies that might include probiotics as well as dietary and lifestyle interventions.

## Conclusions

The microbiome can be defined as all microorganisms that inhabit humans and their interactions with the environment. The findings of studies employing recently developed techniques such as metagenomics as well as those of epidemiological studies indicate that humans are exposed to fewer microorganisms because of changes in factors such as the use of antibiotics and diet, which are accompanied by increasing susceptibility to asthma and allergies. Moreover, these studies illuminate the differences in the microbiomes of healthy people and those with asthma and allergies. Moreover, they show that early exposure to bacteria may protect against these diseases. Further basic studies are required to characterize the lung and the gut microbiomes of healthy people was well as those with asthma and allergies. The ultimate goal is to understand whether aspects of the microbiome are linked to disease and whether manipulation of the microbiome will be useful to preserve lung function, prevent, and treat allergies.
